# The Diversity of Pea Microsymbionts in Various Types of Soils and Their Effects on Plant Host Productivity

**DOI:** 10.1264/jsme2.ME14141

**Published:** 2015-09-15

**Authors:** Jerzy Wielbo, Anna Podleśna, Dominika Kidaj, Janusz Podleśny, Anna Skorupska

**Affiliations:** 1 Department of Genetics and Microbiology, Maria Curie-Skłodowska University, Akademicka 19 str., 20–033 Lublin, Poland; 2 Institute of Soil Science and Plant Cultivation—State Research Institute, Czartoryskich 8 str., 24–100 Puławy, Poland

**Keywords:** Rhizobial populations, biodiversity, symbiotic effectiveness

## Abstract

The growth and yield of peas cultivated on eight different soils, as well as the diversity of pea microsymbionts derived from these soils were investigated in the present study. The experimental plot was composed of soils that were transferred from different parts of Poland more than a century ago. The soils were located in direct vicinity of each other in the experimental plot. All soils examined contained pea microsymbionts, which were suggested to belong to *Rhizobium leguminosarum* sv. *viciae* based on the nucleotide sequence of the partial 16S rRNA gene. PCR-RFLP analyses of the 16S-23S rRNA gene ITS region and *nodD* alleles revealed the presence of numerous and diversified groups of pea microsymbionts and some similarities between the tested populations, which may have been the result of the spread or displacement of strains. However, most populations retained their own genetic distinction, which may have been related to the type of soil. Most of the tested populations comprised low-effective strains for the promotion of pea growth. No relationships were found between the characteristics of soil and symbiotic effectiveness of rhizobial populations; however, better seed yield was obtained for soil with medium biological productivity inhabited by high-effective rhizobial populations than for soil with high agricultural quality containing medium-quality pea microsymbionts, and these results showed the importance of symbiosis for plant hosts.

Rhizobia are soil bacteria that participate in the biological nitrogen fixation process as symbionts of legumes. Symbiosis plays a crucial role in numerous ecosystems because the estimated amount of symbiotically reduced N_2_ is similar to the total amount of N reduced industrially in fertilizer production (up to 70 and 90 Tg N year^−1^, respectively) (8, 24). Individual plant species have different abilities to assimilate symbiosis-derived nitrogen (23), with a large part of individual populations potentially being comprised of low-effective strains (28); however, symbiotically reduced N_2_ may account for more than half of plant nitrogen requirements (8, 14), which may be very advantageous in nitrogen-scarce environments.

Rhizobial populations residing in soils are dynamic and continuously evolving communities. Their diversity stems from the size and plasticity of rhizobial genomes (3, 13), which enables bacteria to adapt to different (soil and endo-symbiotic) habitats. Rhizobia often spread from their initial habitats (19); however, the success of their introduction into new environments relies upon their ability to adapt to numerous biotic and abiotic factors (22). Strain competition and co-habitation in the vicinity enables the horizontal transfer of genetic material between bacteria (2, 10), which may enrich the genetic pool of individual strains and increase intrinsic population diversity. All these variables may completely change the initial structure of the microbial population; therefore, local rhizobial populations may greatly differ from each other (1, 18, 21).

*Rhizobium*-legume symbioses are species-specific, and each host plant may be nodulated by one or a few microsymbiont species (23). Although nodules of the pea (*Pisum*) as well as vetch or bean (*Vicia*) are mostly colonized by *Rhizobium leguminosarum* sv. *viciae* (*Rlv*), other *Rlv*-related rhizobial species were recently reported to nodulate the pea and bean, such as *Rhizobium fabae* and *Rhizobium pisi* (15, 20). The biodiversity of pea microsymbiont populations is known to be site-specific (9, 16). The presence of host plants in the environment (as crops or wild legumes) is favorable for sustaining the genetic diversity of *R. leguminosarum* sv. *viciae* populations, whereas heavy metal pollution of soil or monocropping has been suggested to reduce biodiversity (10, 12, 26). Pea microsymbionts are common inhabitants of Polish soils (12) and their local population may be diverse; thus, individual pea plants may enter into symbiotic interactions with many rhizobial strains (29).

We have used the pea as a “trap plant” to sample some soil-borne rhizobial populations, and have compared their genetic markers. Every soil used in this study was geographically isolated over a century ago, and, at the end of the 19^th^ century (1881), they were moved into one site (Institute of Soil Science and Plant Cultivation, Puławy, Poland) (Fig. 1A), in which they have been exploited in agricultural field experiments. These soils have been placed in plots separated from each other, but located in direct vicinity; therefore, the occasional transfer of microbes (such as by wind, humans, and farming tools) among introduced soils and surrounding (indigenous) soil may have resulted in the mixing of populations including rhizobial populations. In the present study, we assessed the populations of pea microsymbionts in these soils by PCR-RFLP analyses of the 16S-23S rRNA gene ITS region and *nodD* alleles in the populations. The results obtained may assist in determining whether these populations are still different or have been unified due to being in the vicinity of each other for more than a century.

## Materials and Methods

### Soils

The experimental site was located in the Institute of Soil Science and Plant Cultivation, Puławy, Poland, between 51.414635N, 21.959931E and 51.414973N, 21.959536E. It was composed of eight plots, which were established in 1881 and slightly modified in subsequent years. Each plot (14 m^2^) was 1 m deep and surrounded by wooden walls (now made of concrete). Plots were filled with profiles representing common soil types occurring in Poland with different qualities (Table 1).

The plots have always been cultivated by hand tillage in the same manner, received the same fertilization, and the same plant species (mostly cereals, sporadically legumes for green manure) have been simultaneously grown in all plots (5). The soil surrounding the microplots (indigenous soil) was Haplic Cambisol (Dystric)— slightly loamy sand—and was named “soil 9”. Since this indigenous soil could not be cultivated in the same manner as the eight other microplots, it was not used in plant experiments. It acted as a source of bacteria for biodiversity studies on rhizobial populations.

### Enumeration of pea microsymbionts in soils

The number of pea microsymbionts in soils was determined by the most probable number (MPN) method. Briefly, pea plants (*Pisum sativum* cv. Medal) grown from surface-sterilized seeds were cultivated in small plastic pots filled with 200 g of sterilized sand fertilized with 50 mL of Fahraeus N-free medium (27) (one plant per pot). After their emergence, seedlings were inoculated with 1 mL of ten-fold soil dilutions. Six dilution steps (10^−1^–10^−6^) and three replicated pots for each dilution were used. Plants were watered three times a week with sterile water and maintained at 19°C for 10 h (night) and 24°C for 14 h (day). After four weeks, plants were inspected for the presence of root nodules. The total number of positive rhizobial infections was scored for each dilution, and the most probable number of pea microsymbionts in each soil studied was estimated using specialist tables (11). The obtained number was defined as a pea-nodulating unit (PNU) in this study.

### Field experiments

Experiments were conducted in a completely randomized design in triplicate: three 3 m^2^ “microplots” were established on each of the previously described plots. Pea seeds (*Pisum sativum* cv. Medal), 100 seeds (m^2^)^−1^, were sown at the beginning of April (2011), and almost 900 plants (100 seeds × 3 m^2^ × 3 replicas) were considered to be present on each plot. Before sowing, seeds were treated with a commercial fungicide Vitavax 200FS following the instructions of the manufacturer (400 g of the fungicide and 0.4 L of water 100 kg^−1^ of seeds). At flowering (beginning of June), random plants (50 individuals from microplots located on each soil) were harvested for root nodule counting, chlorophyll extraction, and the isolation of rhizobia from nodules. All other (potentially more than 800) plants were harvested at full maturity (middle of July) to evaluate yields by the weights of seeds and straw. The number of seeds per plant was determined using 50 plants randomly selected from the harvested group.

### Bacterial strains

**Fig. 1:**
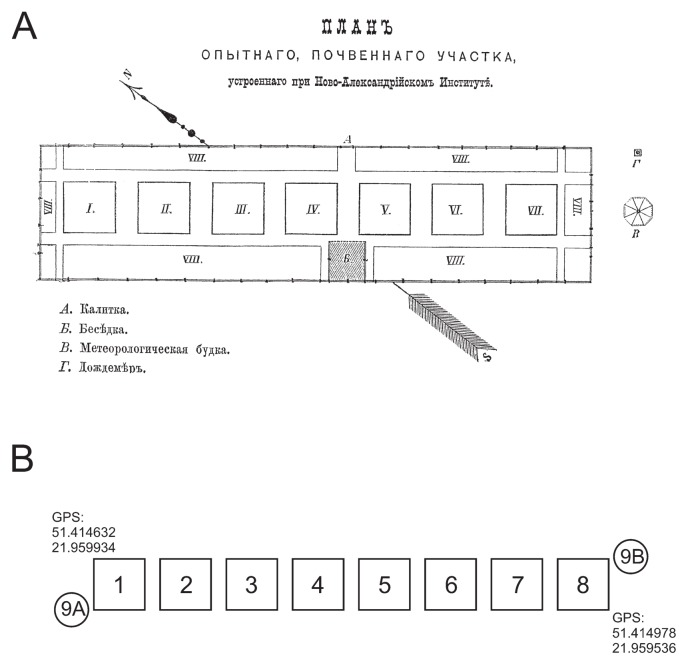
(A) Map of plots published in 1896 in a study entitled “Historical note about the construction of experimental plots at Institute of Nova Alexandria”. This study was written in Russian because, at the end of 19^th^ century, this region of Poland was a part of the Russian Empire; at this time, the town “Puławy” was called “Nova Alexandria”. Map title: Plan of experimental plots constructed at the Institute of Nova Alexandria; abbreviations: A–wicket, Ƃ–arbour, B–meteorological box, Γ–rain gauge. (B) Present plan of plots; abbreviations: 1, 2, …, 8–soil 1, soil 2, …, soil 8; 9A–indigenous soil, sampling site “A”, 9B–indigenous soil, sampling site “B”.

Rhizobial strains were isolated from the nodules of flowering peas grown on eight plots with different soils. Seventy-three strains were isolated from the nodules of pea plants grown in the vicinity on soil surrounding the plots (soil 9). In this case, rhizobia were sampled from the nodules of peas grown on two sites (named “9A” and “9B”, Fig. 1B) flanking experimental plots, and were then coupled together in one group. Pea nodules were surface-sterilized, crushed, and their content was plated on 79CA medium (27). Plates were incubated for 2 d at 28°C. Isolates were purified by successive re-streaking of single colonies and pure cultures were used in further experiments. A total of 680 strains were obtained, *i.e.* 80, 82, 79, 78, 85, 69, 72, 62, and 73 from soils 1 to 9, respectively.

### DNA analyses

Genomic DNA was purified from the isolates as well as from reference strain *Rhizobium leguminosarum* sv. *Viciae* 3841 (30), and PCR-RFLP assays of the 16S-23S rRNA gene ITS region were carried out using primers: FGPS1490 (5′-TGCGGCTGGATCACCTCCTT-3′) and FGPL132 (5′-CCGGGTTTCCCCATTCGG-3′). A PCR-RFLP analysis of the *nodD* gene was conducted using primers: NBA12-5′ (5′-GGATSGCAATCATCTAYRGMRTGG-3′) and NBF12-5′ (5′-GGATCRAAAGCATCCRCASTATGG-3′) (18). Amplicons were digested with *Bsu*RI and *Taq*I (16S-23S rRNA gene ITS region) or *Bsu*RI (*nodD*) restriction endonucleases and separated by 3% agarose gel electrophoresis.

### Pot experiment

Experiments were conducted in the greenhouse of the Institute of Soil Science and Plant Cultivation—State Research Institute in Puławy between 2012 and 2013. Mitscherlich pots were filled with 0.65 kg of perlite and fertilized with 300 mL of Fahraeus N-free medium (27). Ten pea seeds (*Pisum sativum* cv. Medal) were sown in each pot, some seedlings were randomly thinned after the phase of full emergence (*i.e.* 15 d after sowing), and five plants were then cultivated in each pot. Each experimental group consisted of three pots.

Plants were inoculated with a mixture of bacterial strains prepared as follows: rhizobial strains were grown overnight in liquid TY medium at 28°C, and absorbance at 550 nm of the cultures was set to 0.2 by dilution in liquid TY to obtain bacterial suspensions with equal numbers of cells for each strain. Five-milliliter aliquots of all cultures derived from the same soil were integrated into one suspension, which was centrifuged, the supernatant was discarded, and the bacterial pellet was suspended in sterile water. The optical densities of all (*i.e.* eight) suspensions were equalized (OD_550_ = 0.2), and 10 mL of the suspension (~7×10^9^ bacteria) was used to inoculate one of the pots.

The plants were watered using demineralized water twice a day (by a gravimetric method) and the humidity in pots was maintained at 40% of the maximum water-holding capacity. The harvest of plants was performed at the phase of the end of flowering (BBCH 69). The experiment was repeated twice, and the mean of these experiments is presented.

## Results

### The diversity of the 16-23S rRNA gene ITS region in pea microsymbionts

The number of pea microsymbionts differed markedly among the soils studied. The largest rhizobial populations were found in soil 1 and in soil 9 surrounding the plots (approximately 1.5×10^6^ PNU (g soil)^−1^), in contrast to only 1.5×10^2^ and 4.2×10^2^ PNU (g soil)^−1^ in soils 6 and 8, respectively. In the other soils, the number of pea microsymbionts was moderate: 1.5×10^5^; 9.2×10^3^; 1.5×10^3^; 4.2×10^4^, and 9.2×10^3^ PNU (g soil)^−1^ for soil 2, soil 3, soil 4, soil 5, and soil 7, respectively (Table 1).

**Fig. 2:**
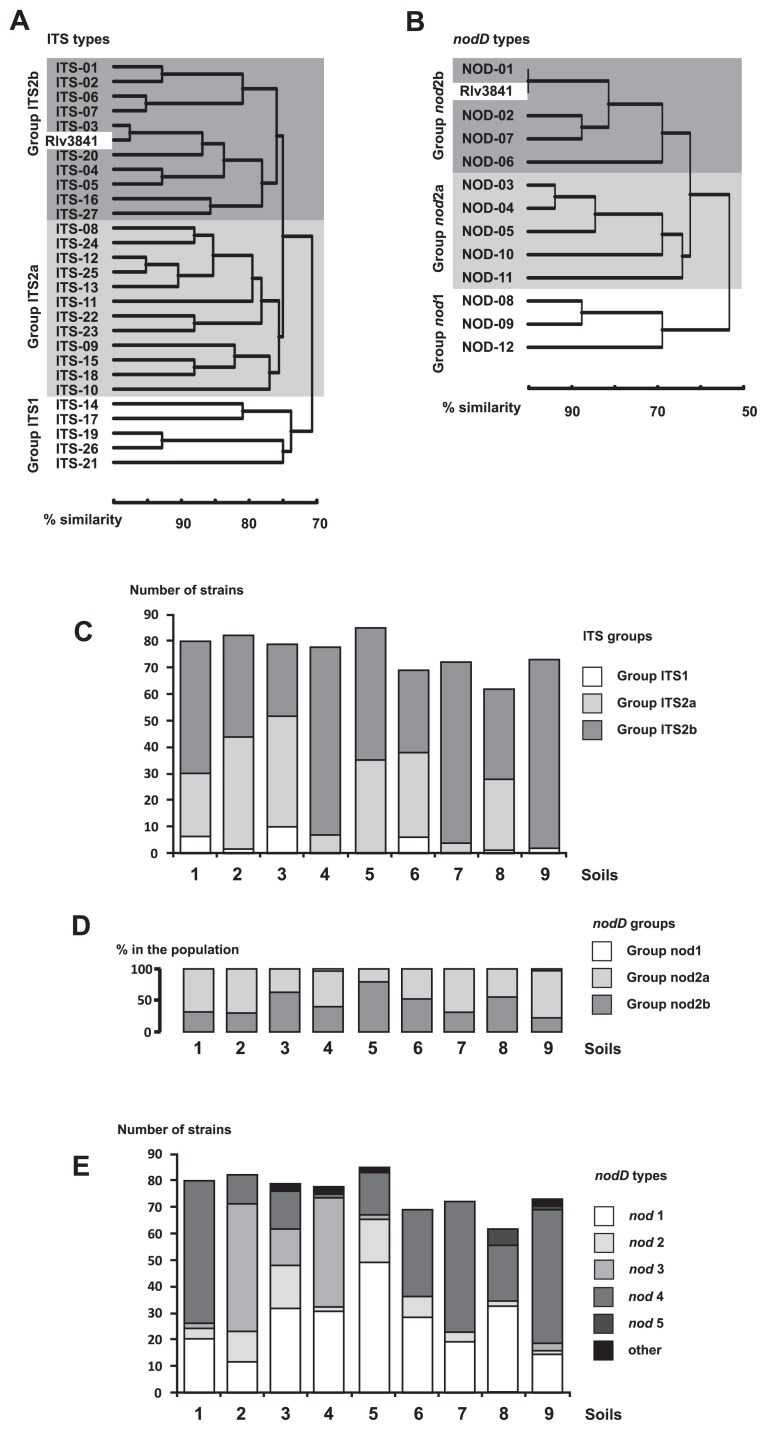
(A) Dendrogram of genetic relationships among rhizobial pea nodule isolates obtained on the basis of PCR-RFLP patterns of the 16S-23S rRNA gene ITS region. The dendrogram was constructed using the UPGMA clustering method. Rlv3841—*R. leguminosarum* sv. *viciae* (reference strain). (B) The dendrogram of genetic relationships among rhizobial pea nodule isolates obtained on the basis of PCR-RFLP patterns of the *nodD* gene region. The dendrogram was constructed using the UPGMA clustering method. Rlv3841—*R. leguminosarum* sv. *viciae* (reference strain). (C) The number of strains belonging to *ITS* clades in the nine studied rhizobial populations. (D) The percentage of strains belonging to different *nod* clades in the nine studied rhizobial populations. (E) The number of strains belonging to *nod* types in the nine studied rhizobial populations.

Twenty-seven different 16S-23S rRNA gene ITS region profiles were found in the whole set of nodule isolates, including 680 strains, originating from pea plants grown in nine different soils (details not shown). Partial sequencing of the 16S rRNA gene of eighteen randomly selected strains suggested that they belonged to *Rhizobium leguminosarum* (Fig. S2). The dendrogram based on PCR-RFLP analyses (Fig. 2A) demonstrated that the ITS region profiles were dividable into two major clusters exhibiting less than 70% similarity with each other: group 1, containing isolates with the *ITS-14*, *ITS-17*, *ITS-19*, *ITS-21*, and *ITS-26* types (3.5% of strains) and group 2, containing 22 ITS types. In group 2, two subclades were identified, which shared 73% similarity: group 2a (12 types of ITS, 216 isolates), and group 2b (10 types of ITS, 440 isolates). The highest levels of similarity, over 95%, were found in the pairs *ITS-06*/*ITS-07* and *ITS-12*/*ITS-25* (Fig. 2A). The PCR-RFLP pattern of the reference strain *R. leguminosarum* sv. *Viciae* 3841 was included in group 2b, and its closest relative was *ITS-03*. The strains belonging to group 1 were not numerous, but detectable in two Haplic Cambisols (Dystric) and Fluvic Cambisol (soils 1, 3, and 6). The strains belonging to group 2b outnumbered other groups in Haplic Luvisol (soil 4), Haplic Cambisol (Eutric) (soil 7), and indigenous soil (soil 9). The strains of group 2a accounted for a large component of pea microsymbiont populations in most soils (*i.e.*, soils 1, 2, 3, 5, 6, and 8) (Fig. 2C).

The frequency of the occurrence of strains belonging to the defined ITS groups differed markedly between the soils studied. The most prevailing and widespread was *ITS-01*, which was found in all types of soils, and the total number of isolates with this profile reached 148. The other extremity was occupied by profiles *ITS-20*, *ITS-23*, *ITS-24*, *ITS-25*, and *ITS-26*, which were found as individuals. Moreover, some ITS types such as *ITS-02*, *ITS-04*, *ITS-05*, and *ITS-14* appeared to be soil-specific: 82% of *ITS-02* profiles were found in soil 4, 72% of rhizobia with *ITS-04*, and 83% with *ITS-05* were found in soil 5, while 91% of strains belonging to the *ITS-14* group originated from soil 3.

### The diversity of the *nodD* gene in pea microsymbionts

The genetic diversity of the symbiotic region in pea nodule isolates was evaluated on the basis of polymorphisms in the *nodD* regulatory gene. Twelve distinctive patterns of *nodD* PCR-RFLP were found in the 9 rhizobial populations studied (details not shown), and were divided into two clusters sharing less than 50% similarity with each other: group 2 contained 99.56% of isolates and group 1 included only 0.44% of isolates (Fig. 2B). Two subclades were identified in group 2, and shared 47% similarity: group 2a (isolates with the profiles of *nod-03*, *nod-04*, *nod-05*, *nod-10*, and *nod-11*) and group 2b (isolates with the profiles of *nod-01*, that of the reference strain *R. leguminosarum* sv. *viciae* 3841, *nod-02*, *nod-06*, and *nod-07*). Strains belonging to group 2b definitely prevailed in populations derived from soil 3 and soil 5, whereas populations derived from soils 1, 2, 7, and 9 were clearly outnumbered by strains belonging to group 2a (Fig. 2E).

An analysis of the distribution of individual *nodD* profiles revealed that two, *nod-01* and *nod-04*, were widespread and numerous (241 and 295 strains, respectively), two others, *nod-02* and *nod-03*, were less numerous (64 and 65 strains, respectively), and *nod-05* was sporadic (8 strains). The remaining identified *nodD* types (*nod-07* to *nod-12*) were found as single instances. *Nod-02*, *nod-03*, and *nod-05* appeared to be soil-specific because most strains with those nod profiles accumulated in Haplic Leptosol, Fluvic Cambisol, Haplic Chernozem, and one of the Haplic Cambisols (Eutric) (soils 2, 3, 5, and 8, respectively) (Fig. 2D).

### Evaluation of population diversity

**Fig. 3:**
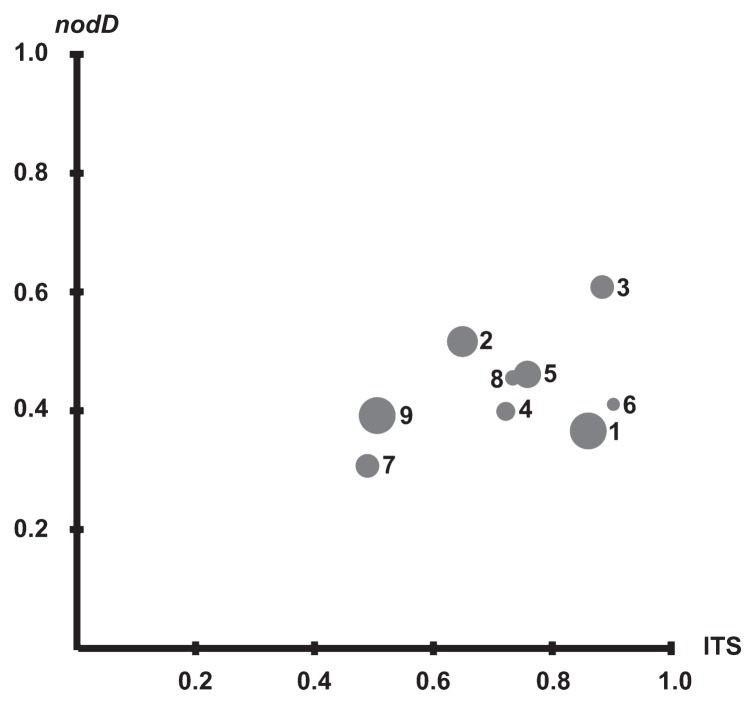
Shannon-Wiener index values calculated for studied rhizobial populations on the basis of the number of strains belonging to different *ITS* classes (X axis) and the number of strains belonging to different *nod* classes (Y axis) compared with the size of populations. Each population is symbolized by an individual dot, and the numbers on the plot (1, 2, …, 9) refer to soil numbers (soil 1, soil 2, …, soil 9). The diameters of dots are proportional to the log of the number of pea microsymbionts per g of soil.

The populations of pea microsymbionts in soils differed not only in their size (Table 1), but also in their diversity. The Shannon-Wiener ecological diversity index, which was determined using the results of the PCR-RFLP analysis of the 16S-23S rRNA gene ITS region, varied from 0.486 (soil 7) to 0.892 (soil 6), but varied from 0.320 (soil 7) to 0.609 (soil 3) using the results of the PCR-RFLP analysis of the *no* gene region. Although no relationship was observed between the diversity and size of the population, some low-diversified (derived from soil 7 or 9) or high-diversified (derived from soil 3) pea microsymbiont populations were indicated (Fig. 3).

Bray-Curtis dissimilarity index values calculated on the basis of the number of strains belonging to different ITS classes revealed that populations of pea microsymbionts gathered into groups, *e.g.* the soil 1/soil 6/soil 8 group and the soil 4/soil 7/soil 9 group (Table 2). On the other hand, Bray-Curtis dissimilarity index values calculated on the basis of the number of *nodD* types in the populations studied showed that the most similar groups of rhizobia were populations originating from soils 1, 7, and 9 (Table 2). The most “dissimilar” population, differing in ITS as well as *nodD* alleles, was isolated from Haplic Leptosol (soil 2).

### Symbiotic properties of natural and reconstituted rhizobial populations

The types of soils markedly affected plant growth, nodulation, and plant yield. Pea plants grown on Haplic Luvisol (soil 4) formed 7.25-fold more nodules, the fresh mass of which was 7.17-fold higher than that of pea plants grown on Haplic Cambisol (Dystric) (soil 6) (Table 3). However, no correlation was observed between the number of rhizobia in soil and number of nodules or wet mass of nodules. Differences in plant nodulation at flowering were followed by differences in the pea yield at full plant maturity. Pea plants grown on Haplic Luvisol (soil 4) had the highest nodule number and weight, chlorophyll content, seed number, and weight of seed and straw among the tested soils (Table 3). They surpassed values obtained on Haplic Cambisol (Dystric) (soil 6) by 2.8-fold, 7.6-fold, and 5.9-fold for seed number, seed yield, and straw yield, respectively (Table 3).

Seed number per plant did not correlate with the number of nodules, whereas a positive correlation was observed between the nodule number and seed yield as well as straw yield (0.876 and 0.863, respectively, both at *p*<0.01). Seed and straw yields also positively correlated with the fresh mass of nodules (0.913 and 0.903, respectively, both at *p*<0.005). No significant differences were observed in the chlorophyll content in the leaves of plants; however, chlorophyll A content at flowering positively correlated with straw yield at full plant maturity (0.718, *p*<0.05).

The symbiotic properties of rhizobial populations reconstituted with pea microsymbionts isolated from each soil were assessed in additional pot experiments. Reconstituted populations (considered as “representatives of soil populations”) contained a similar number of rhizobial cells, but were composed of different number of strains, from 62 (soil 8) to 85 (soil 4). In this experiment, we focused exclusively on the effects of rhizobial populations on plant growth. Reconstituted populations, originating from Haplic Luvisol (soil 4) and Haplic Cambisol (Eutric) (soil 7), significantly promoted the growth of peas, increasing the dry mass of shoots by 41.6 and 32.9%, respectively, from that in uninfected plants. On the other hand, those from Haplic Cambisol (Dystric), Haplic Leptosol (Calcaric), and Fluvic Cambisol (soils 1, 2, and 3, respectively) did not exert positive effects on the growth of the overground part of peas even though they nodulated the plant host (Table 4). Only one rhizobial population, originating from Haplic Chernozem (soil 5), significantly increased the dry mass of infected roots. Marked differences were observed in the number of nodules between groups.

## Discussion

The rhizobial populations examined in the present study differed markedly in their size, genetic structure, and promotion of plant growth. Most of the soils used in this study were rich in rhizobia, possible due to occasional legume cultivation (5); however, the number of pea microsymbionts in the eight soils studied markedly varied, by up to 10^4^–fold, in the range of 1.5×10^2^ to 1.5×10^6^ PNU (g soil)^−1^, with the lowest number of pea microsymbionts being found in two sandy soils (soil 6 and soil 8). This may have been the result of low pH and/or occasional droughts, both of which are considered to be factors that reduce the number of soil rhizobia (12, 22). On the other hand, since soil 1, one of three low-pH soils, was inhabited by numerous pea microsymbionts, low pH and/or droughts cannot be the sole factors decreasing the number of rhizobia in these soils. Despite the presence of a large “rhizobial reservoir” in the soil surrounding the plots (PNU [g soil]^−1^), more than half of the soils used in this study contained markedly smaller rhizobial populations than that in soil 9. This result suggests the influence of factors that do not favor rhizobial proliferation and persistence in individual plots.

A comparison of Bray-Curtis dissimilarity index values obtained for 16S-23S rRNA gene ITS-based analyses, as well as *nodD*-based analyses revealed that, in both cases, soils 2, 3, and 5 were the most dissimilar to soil 9; therefore, we speculated that these soils retained the highest number of “autochthonous pea microsymbionts”. On the other hand, the lowest dissimilarity index values (0.090 and 0.200 for the 16S-23S rRNA gene ITS region and *nodD* region, respectively) was calculated for the pair soil 9—soil 7, which suggested the marked uniformity of these populations.

A comparison of the distribution of ITS and/or *nodD* classes in the populations studied allowed for the identification of some ITS and/or *nodD* types that were widespread among the analyzed soils. For example, *ITS-01* was the dominant ITS type in indigenous soil 9. Furthermore, it constituted a large part of population in some of the soils examined, and, thus, may have been a consequence of the invasion of autochthonous strains (derived from soil 9) with *ITS-01* into soils introduced into this environment. It is important to note the overall similarity in the 16S-23S rRNA gene ITS region allele frequency and *nodD* allele distribution between soils 7 and 9. If the hypothesis of rhizobial invasion from indigenous soil 9 into other soils is plausible, this may have been a result of the replacement of the initial rhizobial population of soil 7 by influx strains derived from soil 9.

Some soil-specific 16S-23S rRNA gene ITS region alleles, such as *ITS-02*, *ITS-04*, *ITS-05*, *ITS-09*, *ITS-10*, and *ITS-14*, were detected in one or two populations, and were not found in strains isolated from soil 9. Similar soil-specific patterns of strain occurrence were recorded for *nod-2* and *nod-3* alleles; therefore, we speculated that these alleles were present in the “initial” populations and still persist in the soils. The rhizobial populations examined differed not only in their strain composition, but also in their potential for promoting plant growth. As shown in the pot experiments, three of the populations studied were ineffective despite the presence of nodules on pea roots. Although this was unusual, field studies demonstrated that most indigenous rhizobial populations were low-effective species (6). Rhizobia that do not reduce atmospheric nitrogen for its host do not have to be “useless” for plants because they have been suggested to act as PGPR bacteria responsible for phosphate acquisition, competition with pathogens, or eliciting plant defense reactions (25). Therefore, they may be beneficial for plant hosts living in the soil, and this effect may not have manifested under our experimental conditions.

Field experiments enabled us to standardize some of the environmental variables, for example, climate- and management-related factors, which were the same due to the localization of all experimental fields in one place. However, the growth of plants on plots may be affected by different soil-borne factors, such as physical and chemical soil properties, and overall microbial activity. In contrast, in the pot experiments, plant growth was mainly dependent on one variable, *i.e.* the symbiotic activity of the strains. Despite these differences in the experimental design, two of the best scores for pea growth and yield were obtained for peas grown on soils 4 and 7, and these two types of soils were also the source of rhizobial populations that were the most active in promoting plant growth in the pot experiment. This result confirmed the value of these two populations and the importance of symbiosis in plant host growth.

Ample plant growth was also observed in soil 5. The Haplic Chernozem had the best index of soil quality, and the chlorophyll content of pea plants grown on soil 5 suggested that these plants were supplied with sufficient amounts of N, P, and K (4); however, the yield of peas obtained for soil 5 was lower than that obtained for soils 4 and 7. Symbiotic nitrogen fixation is known to be more costly for plant hosts than mineral/organic N acquisition from the soil (17); therefore, the precise mechanisms underlying autoregulation suppressing nodulation at high soil N availability evolved (7). However, since symbiotic nitrogen fixation may be a “more stable” nitrogen source, available during the whole period of plant growth, it may be favorable for obtaining a better seed yield in further perspectives.

## Supplementary Information



## Figures and Tables

**Table 1:** Soil characteristics in eight experimental plots

Soil no.	Soil type	pH (in KCl)	Total N (%)	Humus (%)	PNU (g soil)^−1^
1	Haplic Cambisol (Dystric)—light loamy sand	4.70	0.055	1.17	1.5×10^6^
2	Haplic Leptosol (Calcaric)—heavy loamy sand	7.78	0.106	2.11	1.5×10^5^
3	Fluvic Cambisol—medium loam	7.62	0.089	1.63	9.2×10^3^
4	Haplic Luvisol—very fine sand	5.74	0.077	1.46	1.5×10^3^
5	Haplic Chernozem—light silty loam	7.67	0.207	3.70	4.2×10^4^
6	Haplic Cambisol (Dystric)—slightly loamy sand	4.81	0.050	1.11	1.5×10^2^
7	Haplic Cambisol (Eutric)—very fine sand	5.82	0.089	1.60	9.2×10^3^
8	Haplic Cambisol (Eutric)—light loamy sand	4.93	0.084	1.72	4.2×10^2^

**Table 2:** Bray-Curtis dissimilarity index values calculated on the basis of the number of strains belonging to different *ITS* classes and *nodD* classes in pea microsymbiont populations studied

	ITS										
*nodD*		Soil 1	Soil 2	Soil 3	Soil 4	Soil 5	Soil 6	Soil 7	Soil 8	Soil 9	ITS
	Soil 1		0.889	0.912	0.544	0.927	0.383	0.618	0.282	0.503	
	Soil 2	0.654		0.466	0.913	0.545	0.841	0.961	0.944	0.871	
	Soil 3	0.654	0.366		0.911	0.390	0.878	0.960	0.929	0.882	
	Soil 4	0.654	0.366	0.414		0.902	0.646	0.333	0.643	0.338	
	Soil 5	0.654	0.366	0.414	0.411		0.831	0.949	0.973	0.873	
	Soil 6	0.654	0.366	0.414	0.411	0.325		0.702	0.359	0.577	
	Soil 7	0.654	0.366	0.414	0.411	0.325	0.220		0.716	0.200	
	Soil 8	0.654	0.366	0.414	0.411	0.325	0.220	0.358		0.615	
	Soil 9	0.654	0.366	0.414	0.411	0.325	0.220	0.358	0.422		
	*nodD*										

**Table 3:** Nodulation and chlorophyll content in leaves of peas cultivated on eight different soils and harvested at flowering (BBCH 65), as well as seed number and pea yield for plants harvested at full maturity (BBCH 89)

Soil	Plants harvested at BBCH 65				Plants harvested at BBCH 89		
	
	Nodule number per plant	Fresh mass of nodules g per plant	Chlorophyll content mg (g)^−1^ fresh mass		Straw yield g (m^2^)^−1^	Seed yield g (m^2^)^−1^	Seed number per plant

			Chlorophyll A	Chlorophyll B			
1	49 ± 3	0.32 ± 0.06	2.40 ± 0.24	1.52 ± 0.23	328 ± 14	525 ± 14	15.9 ± 0.9
2	45 ± 4	0.20 ± 0.08	2.38 ± 0.33	1.46 ± 0.28	214 ± 9	239 ± 9	7.5 ± 0.3
3	33 ± 2	0.17 ± 0.04	2.46 ± 0.25	1.67 ± 0.32	267 ± 7	338 ± 12	11.5 ± 0.5
4	87 ± 7	0.43 ± 0.11	2.67 ± 0.16	2.04 ± 0.35	745 ± 7	1109 ± 23	18.2 ± 0.5
5	64 ± 8	0.41 ± 0.04	2.77 ± 0.13	2.36 ± 0.29	484 ± 11	693 ± 15	10.2 ± 0.9
6	12 ± 3	0.06 ± 0.01	2.48 ± 0.33	1.81 ± 0.34	126 ± 7	142 ± 8	6.5 ± 0.7
7	46 ± 2	0.36 ± 0.05	2.69 ± 0.09	2.02 ± 0.30	512 ± 3	759 ± 8	12.2 ± 0.5
8	36 ± 1	0.30 ± 0.05	2.74 ± 0.14	2.05 ± 0.21	436 ± 8	535 ± 7	12.8 ± 0.6

**Table 4:** Dry mass and nodule numbers of pea plants inoculated with rhizobial populations derived from each soil

Rhizobia derived from	shoot dry mass (g per pot)	Root dry mass (g per pot)	Number of nodules per plant
Soil 1	2.58 ± 0.23^a^	3.08 ± 0.23^a^	57.3 ± 15.0^a^
Soil 2	2.66 ± 0.58^a^	2.78 ± 0.18^a^	38.0 ± 11.2^a^
Soil 3	2.69 ± 0.66^a^	2.92 ± 0.19^a^	45.8 ± 11.4^a^
Soil 4	3.78 ± 0.31^b^	3.24 ± 0.16^a^	67.0 ± 19.2^a^
Soil 5	3.30 ± 0.65^ab^	3.91 ± 0.21^b^	52.8 ± 13.6^a^
Soil 6	3.07 ± 1.68^ab^	3.07 ± 0.19^a^	32.5 ± 11.2^a^
Soil 7	3.55 ± 1.06^b^	3.17 ± 0.26^a^	48.5 ± 16.3^a^
Soil 8	3.26 ± 0.60^ab^	3.24 ± 0.24^a^	53.0 ± 19.7^a^
Control (plants without rhizobia)	2.67 ± 0.18^a^	3.27 ± 0.19^a^	0^b^

a,b values marked with the same letter did not significantly differ at *P*<0.05.

Each experimental group consisted of thirty plants (five plants per pot, three pots per group, two replicas of the experiment).
