# Supplementary material for: The Diversity of Pea Microsymbionts in Various Types of Soils and Their Effects on Plant Host Productivity
Source: Microbes Environ. 2015 Sep 15;30(3):254–61. doi: 10.1264/jsme2.ME14141 (PMC4567564; doi:10.1264/jsme2.ME14141)
Supplement: Supplementary file 1 [file 30_254_s1.pdf]

Table S1

The number of pea microsymbionts in soils used in the experiment and the number of strains belonging to individual ITS groups in the studied rhizobial populations

	Soil 1	Soil 2	Soil 3	Soil 4	Soil 5	Soil 6	Soil 7	Soil 8	Soil 9
Number of rhizobia (PNU (g soil)$^{-1}$)									
	1.5×10^6	1.5×10^5	9.2×10^3	1.5×10^3	4.2×10^4	1.5×10^2	9.2×10^3	4.2×10^2	1.5×10^6
Total number of isolates									
	80	82	79	78	85	69	72	62	73
Number of isolates belonging to different ITS groups									
ITS-01	13	1	1	30	1	6	49	4	43
ITS-02	0	0	0	18	0	0	4	0	0
ITS-03	2	33	19	5	25	6	1	0	7
ITS-04	0	1	3	0	13	1	0	0	0
ITS-05	0	1	0	0	10	1	0	0	0
ITS-06	21	1	0	7	0	13	9	15	14
ITS-07	11	0	0	11	1	3	5	15	7
ITS-08	19	0	1	3	0	24	0	20	1
ITS-09	0	4	21	0	23	0	0	0	0
ITS-10	1	32	10	0	0	0	0	1	0
ITS-11	0	5	6	0	9	0	0	0	0
ITS-12	0	0	0	4	1	2	3	0	0
ITS-13	0	1	1	0	0	1	0	2	0
ITS-14	1	1	10	0	0	0	0	0	0
ITS-15	3	1	0	0	2	5	0	0	1
ITS-16	0	1	4	0	0	0	0	0	0
ITS-17	0	0	0	0	0	3	0	1	0
ITS-18	1	0	1	0	0	0	1	1	0
ITS-19	4	0	0	0	0	0	0	0	0
ITS-20	0	0	0	0	0	1	0	0	0
ITS-21	0	0	0	0	0	3	0	0	0
ITS-22	0	0	0	0	0	0	0	2	0
ITS-23	0	0	1	0	0	0	0	0	0
ITS-24	0	0	1	0	0	0	0	0	0
ITS-25	0	0	0	0	0	0	0	1	0
ITS-26	1	0	0	0	0	0	0	0	0
ITS-27	3	0	0	0	0	0	0	0	0

Table S2

The number of strains belonging to individual *nod* groups in studied rhizobial populations

	Soil 1	Soil 2	Soil 3	Soil 4	Soil 5	Soil 6	Soil 7	Soil 8	Soil 9
Total number of isolates									
	80	82	79	78	85	69	72	62	73
Number of isolates belonging to different *nod* groups									
nod-01	20	11	34	31	50	29	19	32	15
nod-02	5	14	14	1	17	7	3	2	1
nod-03	1	46	15	0	1	0	0	0	2
nod-04	54	11	14	43	16	33	50	22	52
nod-05	0	0	0	1	0	0	0	6	1
nod-06	0	0	1	0	0	0	0	0	0
nod-07	0	0	1	0	0	0	0	0	0
nod-08	0	0	0	1	0	0	0	0	0
nod-09	0	0	0	1	0	0	0	0	0
nod-10	0	0	0	0	1	0	0	0	0
nod-11	0	0	0	0	0	0	0	0	1
nod-12	0	0	0	0	0	0	0	0	1

Figure S1

PCR-RFLP profiles of (a) amplicons of 16S-23S rRNA gene ITS region digested with *Bsu*RI endonuclease, (b) amplicons of 16S-23S rRNA gene ITS region digested with *Taq*I endonuclease and (c) *nodD* gene region digested with *Bsu*RI endonuclease. Lanes 1-23 (for 16S-23S rRNA gene ITS region digested amplicons) and 1-12 (for *nodD* gene digested amplicons) represents different profiles found in studied isolates; R = *Rhizobium leguminosarum* sv. *viciae* 3841 (reference strain), M = molecular marker (100 bp ladder).

Figure S2

UPGMA based dendrogram constructed with partial sequences (640 bp) of 16S rRNA gene of selected eighteen pea microsymbiont isolates and six reference sequences (*R. leguminosarum*

sv. *viciae* 3841 and five rhizobial type strains: *R. leguminosarum* sv. *viciae* USDA2370, *R. leguminosarum* sv. *trifolii* 14480, *R. etli* CFN42, *R. pisi* DSM30132 and *R. loti* LMG6125). Sequences obtained in this study were registered in GenBank database with accession numbers KP998158-KP998175.

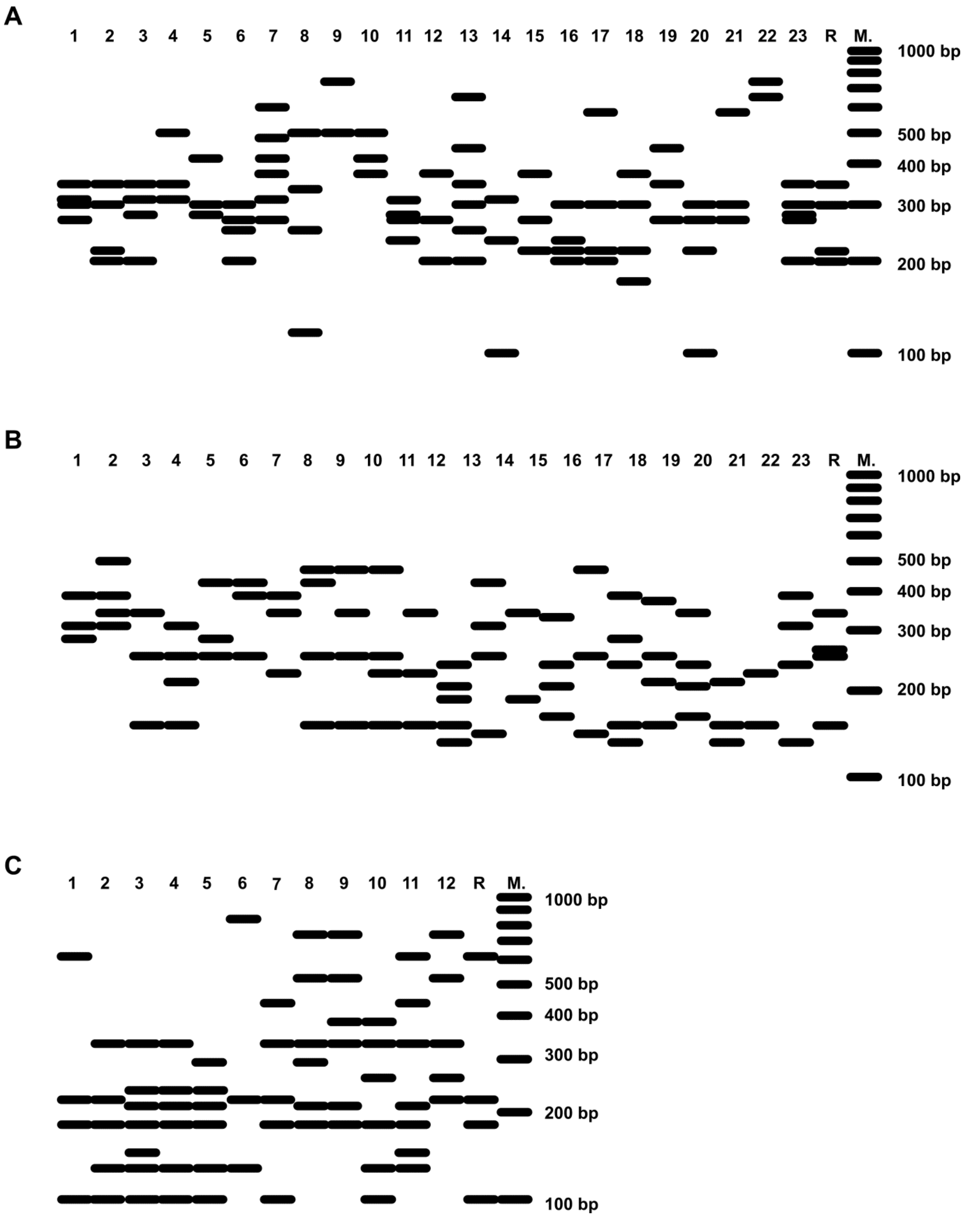
A
1 2 3 4 5 6 7 8 9 10 11 12 13 14 15 16 17 18 19 20 21 22 23 R M.
1000 bp
500 bp
400 bp
300 bp
200 bp
100 bp
B
1 2 3 4 5 6 7 8 9 10 11 12 13 14 15 16 17 18 19 20 21 22 23 R M.
1000 bp
500 bp
400 bp
300 bp
200 bp
100 bp
C
1 2 3 4 5 6 7 8 9 10 11 12 R M.
1000 bp
500 bp
400 bp
300 bp
200 bp
100 bp

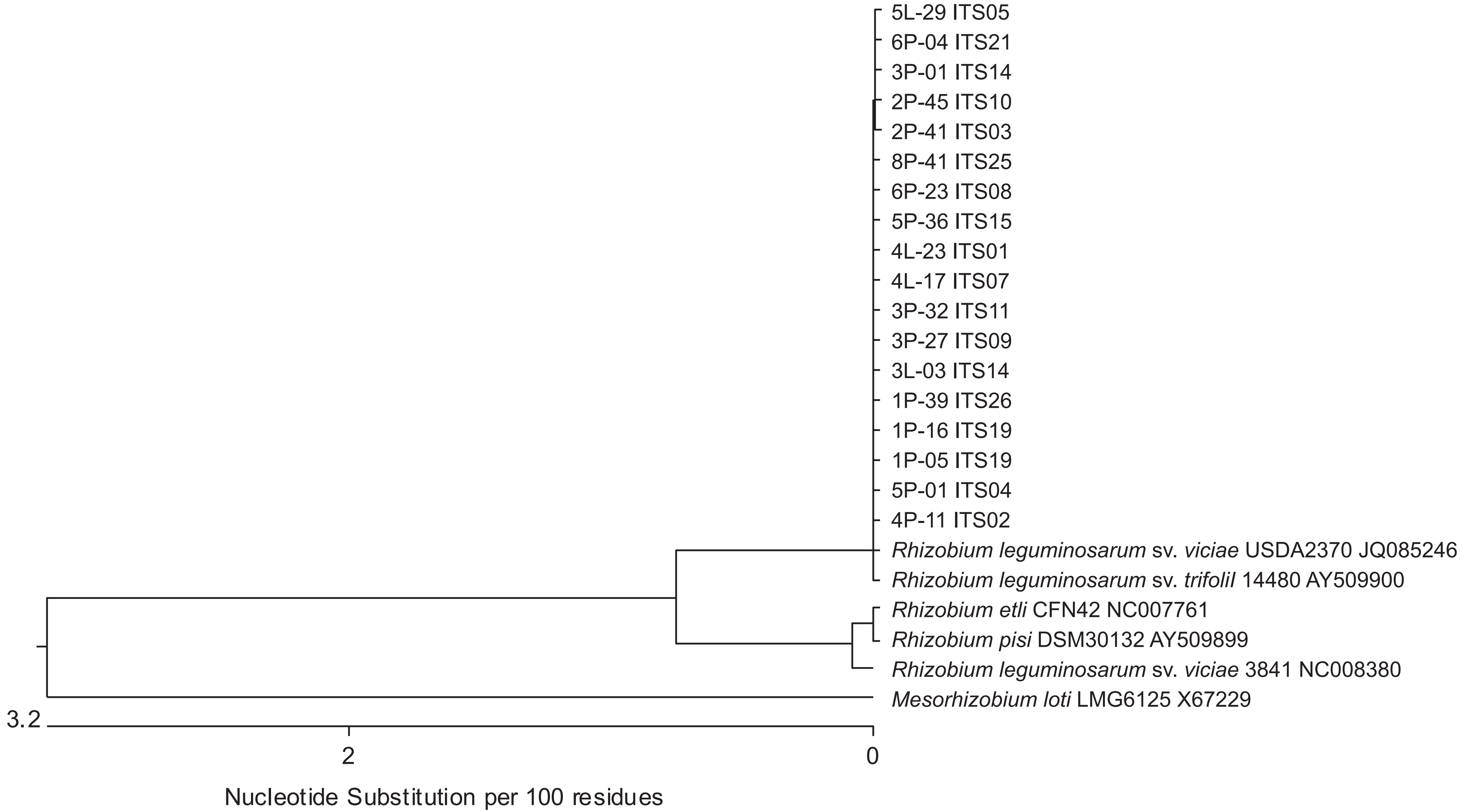
5L-29 ITS05
6P-04 ITS21
3P-01 ITS14
2P-45 ITS10
2P-41 ITS03
8P-41 ITS25
6P-23 ITS08
5P-36 ITS15
4L-23 ITS01
4L-17 ITS07
3P-32 ITS11
3P-27 ITS09
3L-03 ITS14
1P-39 ITS26
1P-16 ITS19
1P-05 ITS19
5P-01 ITS04
4P-11 ITS02
Rhizobium leguminosarum sv. viciae USDA2370 JQ085246
Rhizobium leguminosarum sv. trifolil 14480 AY509900
Rhizobium etli CFN42 NC007761
Rhizobium pisi DSM30132 AY509899
Rhizobium leguminosarum sv. viciae 3841 NC008380
Mesorhizobium loti LMG6125 X67229
3.2
2
0
Nucleotide Substitution per 100 residues
